# Serum peptidomic profiling identifies a minimal residual disease detection and prognostic biomarker for patients with acute leukemia

**DOI:** 10.3892/ol.2013.1574

**Published:** 2013-09-12

**Authors:** WEI SONG, NA WANG, WEI LI, GUANJUN WANG, JIFAN HU, KUN HE, YAN LI, YING MENG, NAIFEI CHEN, SHAOXIN WANG, LINGYUN HU, BIN XU, JIE WANG, AILING LI, JIUWEI CUI

**Affiliations:** 1 Cancer Center, The First Hospital of Jilin University, Changchun, Jilin, P.R. China; 2 Institute of Basic Medical Sciences, National Center of Biomedical Analysis, Beijing, P.R. China; 3 VA Palo Alto Health Care System, Stanford University Medical School, Stanford, CA, USA

**Keywords:** acute leukemia, minimal residual disease, peptide pattern, mass spectrum

## Abstract

The evaluation of minimal residual disease (MRD) in acute leukemia (AL) is currently recognized as a potential critical tool to assess the response and relapse rate of treatments. The present study investigated serum peptides from patients with AL to identify biomarkers that would be useful in providing clinical evaluations and independent prognostic information. The patterns of serum peptides from 123 patients with AL and 49 healthy controls were analyzed using matrix-assisted laser desorption/ionization-time of flight mass spectrometry. Furthermore, diagnostic models of differential peptides were established using the support vector machine (SVM) algorithm to discriminate between the AL patients and healthy controls or between the AL patients with various degrees of remission. Finally, the peptides were applied to evaluate the prognosis of the affected patients. The area under the receiver operating characteristic (ROC) curve (AUC), analyzed using the SVM algorithm to distinguish between the AL patients and healthy controls, was 0.921. The AUC of the models for distinguishing between the newly-diagnosed AL patients and those in AL-hematological complete remission (HCR) and between the AL-HCR patients from those in AL-molecular remission (MR), was 0.824 and 0.919, respectively. A short serum peptide of m/z 4625 was identified to decrease in density in parallel with an increase in the degree of remission, which was used to monitor the MRD level. The intensity of the m/z 4625 peptide was significantly correlated with a poor overall survival (OS). The m/z 4625 peptide was identified to be a partial fragment of SERPINA3. The serum peptide pattern is high in sensitivity and specificity and may be used to discriminate between AL patients with various degrees of remission. The m/z 4625 peptide may be used to monitor the MRD levels and provide independent prognostic information in patients with AL.

## Introduction

Acute leukemia (AL), resulting from the clonal expansion of blasts in bone marrow, is considered to be a clinically, morphologically and genetically heterogeneous disease (1). The rate of complete remission (CR), achieved by intensive chemotherapy, is 50–80% and 80–90% in adult patients with acute myeloid leukemia (AML) and acute lymphoblastic leukemia (ALL), respectively. However, the relapse rate is >60% (2–5), which is the major cause of treatment failure in affected patients. The detection and eradication of minimal residual disease (MRD) is a key issue that merits a controlled clinical evaluation, including optimizing the timing of treatment and guiding decisions with regard to donor lymphocyte infusion post-transplant. Thus, the measure of MRD promises to be an efficient tool for predicting and increasing the survival outcome in AL patients (6–8).

Monitoring MRD levels in patients with AL at various time-points during therapy requires the availability of determination markers. At present, multiparameter flow-cytometry (MFC), polymerase chain reaction (PCR) and fluorescence *in situ* hybridization are useful methods for the detection of MRD in patients with AL. However, the applicability of PCR is restricted to AL with leukemia-specific molecular targets, including promyelocytic leukemia/retinoic acid receptor-α (PML/RARα), FLT3 and T cell receptor (9–12). The widespread applicability of MFC is also complicated by the lack of standardized procedures and a leukemia-associated immunophenotype at the time of diagnosis. Currently, the diagnostic methods mentioned previously are also performed during bone marrow analysis, and patients should undergo bone marrow aspiration under local anesthesia prior to the analysis, which is an invasive and time-consuming procedure. Therefore, the identification of new leukemia markers that are easily detectable and stably expressed in a large proportion of AL cases should simplify the application of MRD studies, aid in extending the benefit to all patients and possibly enhance the sensitivity of MRD detection.

The benefits of serum examination are that it is a minimally invasive and low cost procedure and that serum is easy to acquire and process. Serum protein or peptide levels under a disease state differ from the levels that are present in a healthy state. Thus, serum is a good specimen to be used in disease marker studies. Serum proteomics via the identification of new proteins/peptides have already been successfully performed for tumor diagnosis (13–17). In the field of hematology, the use of the matrix-assisted laser desorption/ionization time of flight mass spectrometry (MALDI-TOF MS) method and complement C3f-desArg and its derivatives has been identified to correlate with MRD levels in patients with PML/RARα-positive acute promyeloid leukemia (APL) (18). These results indicate that human serum peptides, particularly those of low molecular weights, contain important information for tumor diagnosis.

The present study used MALDI-TOF MS analysis to detect specific serum peptidomic biomarkers in order to discriminate between AL patients with various degrees of remission. The specific peptide that may be used to monitor MRD in patients with AL was also identified and its prognostic role was evaluated.

## Materials and methods

### Patients and blood sample preparation

A total of 123 patients with AL and 49 healthy controls were recruited through The First Hospital of Jilin University (Changchun, Jilin, China) between December 2009 and June 2010. The diagnosis of the patients was based on morphology, immunophenotyping, cytogenetics and molecular biology. Among these AL patients, 40 were newly diagnosed, 42 achieved hematological CR (HCR), including 17 M2/M3 patients with cytogenetic abnormalities who reached cytogenetic remission following chemotherapy and 25 patients who achieved consistent remission, including 18 M2/M3 with molecular remission (MR). The remaining AML/ALL patients without cytogenetic/genetic signatures achieved CR for more than one year. A total of 30 patients with newly-diagnosed solid tumors, including lung, breast, liver and colon cancer, and a further 35 patients with benign hematological disorders, including anemia and idiopathic thrombocytopenia (ITP), were also analyzed. The healthy controls were volunteers who underwent a routine physical examination and were confirmed to be in a healthy state by The First Hospital of Jilin University. The clinical characteristics of the patients with AL and the control group are shown in Tables I and II, respectively. Approval for this study was obtained from the Human Subject Committee of The First Hospital of Jilin University, and all patients and healthy controls provided their informed consent.

The serum samples were collected, processed and stored according to standard procedure. Briefly, the serum samples were allowed to clot or sediment at room temperature for 2 h and were then centrifuged at 376 × g for 15 min. The samples were divided into aliquots of 50 μl and stored at −80°C until use. The sera from 10 patients with AL were pooled and analyzed using MALDI-TOF MS. Six within-run assays and six between-run assays were performed to assess the deviation.

### Serum pretreatment using magnetic beads

Copper-chelated magnetic beads and solutions were obtained from the National Center of Biomedical Analysis (Beijing, China) to extract the peptides from the sera. The samples were purified and isolated through binding, washing and elution processes. Briefly, 5 μl beads and 50 μl binding solution were mixed with 5 μl serum. Following a 10-min incubation period, the beads were washed three times using 100 μl washing solution. The bound peptides were then eluted in 20 μl elution solution. A 1 μl elute was mixed with 1 μl CHCA matrix solution, spotted onto target spots (Bruker Daltonik, Bremen, Germany) and dried at room temperature. The peptide calibration standard in the same matrix was applied to the target spots for an external calibration of the instrument. The samples proceeded into MALDI-TOF MS equipped with a pulsed ion extraction ion source.

### MALDI-TOF MS pattern-recognition analysis

All the spectra were analyzed using FlexAnalysis 2.4 software (Bruker Daltonik) to determine the peak m/z values and the intensities in the mass range of 1,000–10,000 Da. To align the spectra, a mass shift of ≤0.1% was determined. The intensities of all the peaks were then normalized to the total m/z ion current of each spectrum using in-house MatchPeaks software and the relative intensity was calculated. P-values for each peak were obtained for their discriminatory power. The support vector machine (SVM) method is an effective algorithm for gene selection and cancer classification and was consequently used for class prediction (http://biosunms.sourceforge.net) (19). The parameters in the Gaussian kernel function were optimized using the grid search approach (20). The models of the training set were built using a selected number of peaks and a five-fold cross-validation scheme.

### Peptide sequence

The identification of peptide m/z 4625 was performed using a nano-liquid chromatography-electrospray ionization-tandem (LC/ESI) MS system. The peptide solutions were loaded onto a C18 trap column (nanoACQUITY; Waters, Milford, MA, USA) at a flow rate of 400 μl/min. Mobile phases A (5% acetonitrile and 0.1% formic acid) and B (95% acetonitrile and 0.1% formic acid) were used for the analytical columns. The gradient elution profile was 5%B-50%B-80%B-80%B-5%B-5%B in 100 min. The eight most intense mono-isotope ions were precursors for collision-induced dissociation. MS/MS spectra were limited to two consecutive scans per precursor ion followed by 60 sec of dynamic exclusion.

### Statistical analysis

The peaks were evaluated using the P-values from a two-tailed t-test in various groups for building the model. The sensitivity, specificity and the area under the receiver operating characteristic (ROC) curve (AUC) were calculated using the validation set data for the three models obtained from the training data. The overall survival (OS) between the two groups with peptide expression differences was estimated using a Kaplan-Meier log rank survival analysis. P<0.05 was considered to indicate a statistically significant difference.

## Results

### Stability and reproducibility of the MALDI-TOF MS spectra

For the reproducibility experiment, a pool of sera from 10 patients with AL was analyzed using the MALDI-TOF MS instrument and used to perform six within-run assays and six between-run assays to observe the deviation. The mean coefficient of variation (CV) of the within- and between-run assays was 8.98% (range, 7.5–11.0%) and 18.2% (range, 13.9–22.3%), respectively, indicating that the analysis of the MALDI-TOF MS spectra was stable and reproducible. The results are shown in Table III.

### Screening peptide patterns to distinguish between AL patients with various degrees of remission

**Figure 1:**
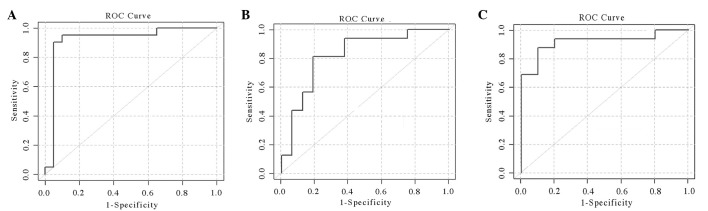
Validation set ROC curves. (A) Distinguishing AL patients from healthy controls (AUC, 0.921). (B) Distinguishing newly-diagnosed AL patients from AL-HCR patients (AUC, 0.824). (C) Distinguishing AL-HCR patients from those achieving deep remission, including M2/M3 patients with MR (AL-MR; AUC, 0.919). ROC, receiver operating characteristic; AL, acute leukemia; AUC, area under the ROC curve; HCR, hematological complete remission; MR, molecular remission.

The serum samples from 123 AL patients and 49 healthy controls were detected for peptide profiling using an MALDI-TOF MS analysis. The samples from 65 AL patients and 29 healthy controls were assigned randomly to a training set and the remaining samples were used as a testing set. Using Flex Analysis 2.4 software (Bruker Daltonics, Bremen, Germany), 249 peaks were discovered in all the training set samples. To identify their discriminatory power, the P-value from each peak was obtained by a two-tailed t-test. The candidate peaks were selected for model building, and 26 peaks were observed to be significantly different between the AL patients and healthy controls (P<0.0001). The SVM algorithm was utilized to generate models and to discriminate the selected 26 peaks as the optimal combination. The detection value of the model was validated using 58 AL patients and 20 healthy controls as the test set. This model distinguished the AL patients from the healthy controls with a sensitivity of 90%, a specificity of 95% and an AUC of 0.921 (Fig. 1A).

As demonstrated in Fig. 1B and C, two patterns of peaks were selected for model building, including 40 newly-diagnosed AL patients, 42 patients with AL-HCR and 25 patients with deep remission (consistent remission for more than one year), which included 18 M2/M3 with molecular remission (MR). A total of 11 peaks (P<0.05) were used to build the model to distinguish between newly-diagnosed AL and AL-HCR patients, which showed the best discriminating power with a sensitivity of 81.25%, specificity of 81.25% and an AUC of 0.824. In the AL-CR and AL-MR models, another 11 peaks (P<0.05) were used. The results of the sensitivity and specificity were 87.5 and 90%, respectively and the AUC was 0.919.

### Screening differential peaks for the evaluation of the MRD

**Figure 2:**
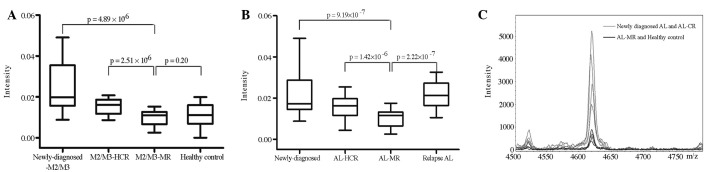
Intensity levels of m/z 4625 peptide in AL and healthy control groups. (A) Boxplot display of m/z 4625 distributions for newly-diagnosed M2/M3, M2/M3-CR, M2/M3-MR and healthy controls. (B) The intensity distribution of m/z 4625 in newly-diagnosed AL, AL-HCR, AL-MR and AL-relapse. The box is bound above and below by the 75th and 25th percentiles and the median is the line within the box. (C) Spectra of the m/z 4625 peptide randomly obtained from newly-diagnosed AL, AL-CR and AL-MR patients and healthy controls. AL, acute leukemia; CR, complete remission; MR, molecular remission; HCR, hematological complete remission.

Serum peptides from 18 M2 patients with AML/eight-twenty-one (ETO) and 35 M3 patients with PML/RARα were evaluated. A significantly different peak with m/z 4625 was identified and the intensity gradually decreased as the remission degree increased. In the healthy controls, the intensity level of m/z 4625 was similar to that of the M2/M3-MR group (Fig. 2A). Notably, for all types of AL patients, the change in m/z 4625 intensity was correlated with the MR/M3 group. Furthermore, the intensity level of m/z 4625 in the relapse group was significantly higher than that of the AL-MR group, including patients in deep remission and M2/M3-MR. There was no difference between the newly-diagnosed AL and relapse AL groups (Fig 2B) or between the newly-diagnosed AL patients and the patients with HCR (Fig. 2C). Among these patients, the intensity level of m/z 4625 in one patient with M1 in HCR was increased. The patient was subsequently followed up and the serum was examined every month. The intensity of the m/z 4625 peak continued to increase and the patient relapsed after three months, with the leukemia cells accounting for 5% of the bone marrow.

### Prognostic value of the m/z 4625 peak

**Figure 3:**
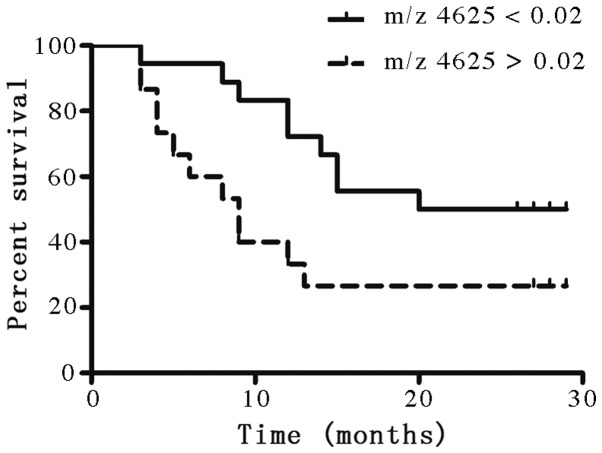
m/z 4625 peptide predicts clinical outcome of newly-diagnosed AL patients. Kaplan-Meier estimates OS of newly-diagnosed patients, who were distributed into two groups. The OS in the group with low m/z 4625 expression (intensity, <0.02) was higher than that of group with high m/z 4625 expression (intensity, ≥0.02). AL, acute leukemia; OS, overall survival.

The second aim of identifying a marker for MRD assessment was to evaluate its role in predicting prognosis. A statistical analysis of the m/z 4625 peak intensity was performed and the peak was observed to be significantly correlated with the prognosis. A total of 33 newly-diagnosed patients possessed complete records. The clinical characteristics of these patients are shown in Table IV. The median follow-up interval was 16 months (range, 3–29 months). The patients were divided into two groups according the intensity of the m/z 4625 peak: Group A displayed an intensity of <0.02 (18 patients) and group B displayed an intensity of >0.02 (15 patients). The features between the two groups were comparable, with the exception of the intensity of the peak. The subjects with intensities of >0.02 showed a poorer OS outcome compared with those with m/z 4625 intensities of <0.02 (P=0.042; Fig. 3).

### Disease specificity of the m/z 4625 peak

**Figure 4:**
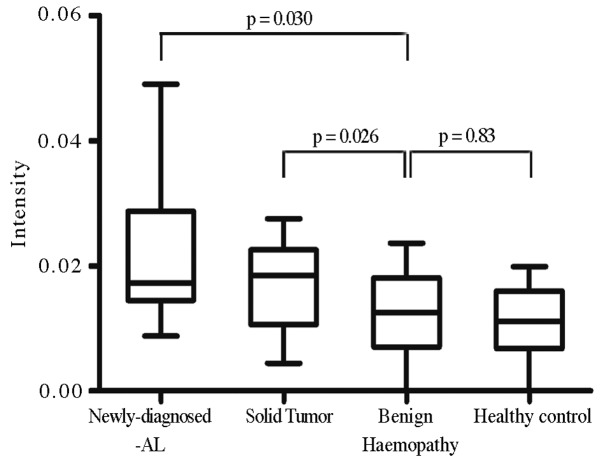
Intensity of the m/z 4625 peptide in other diseases. The intensity of the m/z 4625 peak in the sera of patients with AL or solid tumors was higher than that of patients with benign hematological disorders or healthy controls. AL, acute leukemia.

The intensity of the m/z 4625 peak in the sera of patients with solid tumors and benign hematological disorders was analyzed to confirm whether the peak was restricted to AL patients (Fig. 4). The intensity of m/z 4625 was significantly higher in the malignant diseases, particularly in the AL patients (P=0.014), whereas the level of m/z 4625 intensity in the benign hematological disorders were similar to those of the healthy controls (P=0.587).

### Serum peptide identification

**Figure 5:**
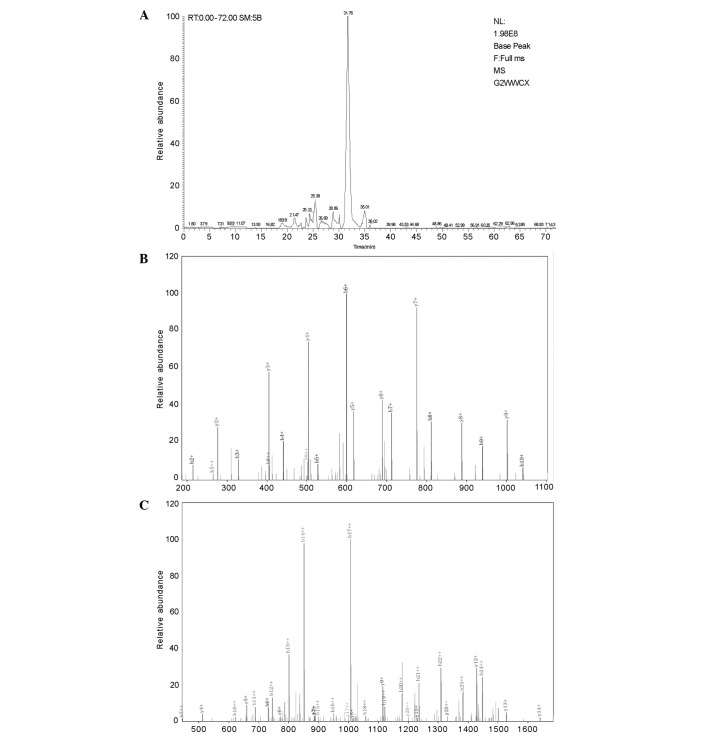
Sequencing results of m/z 4625 using Nano-LC/ESI-MS/MS. A peptide was identified in the sera of the AL patients as a fragment of the SERPINA3 protein. (A) Representative mass spectrum; (B and C) mass spectrum of the ions. LC/ESI-MS, liquid chromatography/electrospray ionization-tandem mass spectometry; AL, acute leukemia.

Using nano-LC/ESI-MS detection, the peptide sequence of 4625 m/z was identified as SAL VETRTIVRFNRPFLMIIVPTDTQNIFFMSKVTNPKQA. A sequence search using the Bioworks Browser 3.3.1 SP1 (Thermo Fisher Scientific, Bremen, Germany) in the International Protein Index database, identified the peptide as a partial fragment of the α-1-antichymotrypsin precursor, SERPINA3 (Fig. 5).

## Discussion

Markers for the detection of the MRD and the prediction of the prognosis of AL play a crucial role in the determination of the treatment regimen and in prolonging the survival of patients. The present study used MALDI-TOF MS to generate profiles of the serum peptides.

The results revealed that the technique system set up in the present study was stable and reproducible. The SVM algorithm was used to distinguish AL patients from healthy controls with a sensitivity of 90% and a specificity of 95%. The peptide pattern was also able to discriminate between AL patients with various degrees of remission. There was a good concordance between the results of this method and conventional methods. These results demonstrate that the serum peptide pattern may reflect the pathological state of the disease and implicate its role in monitoring the MRD of AL.

In the present study, one of the most significantly different peaks with m/z 4625 was identified. The intensity of the peak gradually decreased as the degree of remission increased. In the healthy controls, the intensity of m/z 4625 was similar to that of the AL-MR group. The intensity in the relapse group was significantly higher than that in the AL-MR group, and no difference was observed between the newly-diagnosed and relapsed AL groups. The intensity of the peak in one patient with M1 in HCR was dramatically increased and this patient eventually relapsed within three months. The results indicated that this technique widened the application of MRD detection in AL patients without molecular markers. APL patients in MR showed an increase in the intensity of the peak, which was detected for several consecutive times with various intervals and has been followed up closely since then. It appears that this technique was more sensitive than quantitative PCR.

The intensity of the m/z 4625 peak was observed to significantly correlate with the prognosis of the disease. The patients with a high intensity of the peak at diagnosis displayed a poor prognosis compared with those in the low-intensity group (2-year survival rate 50% vs. 26.7%), implying that the intensity of the peak functions as not only an MRD marker, but also as a prognostic marker. The role of m/z 4625 in prognostic prediction requires further investigation in distinct subtypes of AL.

The sera from patients with solid tumors and benign hematological disorders was analyzed to confirm whether the high intensity of the peak with m/z 4625 was restricted to AL. The intensity of m/z 4625 was significantly higher in malignant diseases, including the solid tumors and particularly in AL (P<0.05). The intensity in the benign hematological disorders was the same as that in the healthy controls, indicating that the m/z 4625 serum peptide may also be used as a diagnostic marker for malignant diseases.

To identify the m/z 4625 peptide, sequencing was performed using nano-LC/ESI-MS/MS, and the peptide was identified as a fragment of SERPINA3. SERPINA3 is an acute phase protein that is produced in the liver, and the concentration may rise during acute and chronic inflammation (21). SERPINA3 also exhibits pro-apoptotic activity (22). Kloth *et al*(23) identified that high SERPINA3 expression correlated significantly with a poor OS in cervical carcinoma using immunohistochemical analysis. In addition, SERPINA3 has been identified to be upregulated in sera from thyroid papillary carcinoma and prostate cancers (24,25). SERPINA3 is involved in promoting the invasion and metastasis of malignant melanomas and cell lines, where it may have a role in regulating apoptosis and invasiveness (26).

In conclusion, the present study indicates that the approach reported here may identify new markers for minimally invasive, fast, universal and sensitive MRD monitoring in AL. The present approach also highlighted the potential of serum peptide alterations as new and useful markers to predict the prognosis of AL patients. Although additional studies are required to draw definite conclusions, the alterations of serum peptide levels may offer an improved understanding of the mechanism involved in the development of AL. Studies aimed at identifying the possible origin of the peptide would be the next investigations to be undertaken.

## Figures and Tables

**Table I:** Clinical characteristics of patients with AL.

	Newly-diagnosed AL		AL-HCR		AL-MR		
				
Acute leukemia	Training set	Test set	Training set	Test set	Training set	Test set	Relapse
Male:female, n	14:10	8:8	14:12	9:7	7:8	4:6	8:8
Age, mean (range), years	37.9 (15–73)	44.6 (18–75)	36.4 (15–56)	33.3 (11–62)	34.8 (13–55)	37.9 (23–55)	33.8 (15–64)
WBC, ×10^9^	27.4	23.4	2.86	5.4	4.5	6.2	40.0
Mean, range	1.2–166.6	0.5–70	0.1–5.7	0.5–17.3	1.3–7.6	3.7–11.2	1.3–197.8
FAB classification, n							
AML	17	13	17	11	13	8	7
ALL	7	3	9	5	2	2	9
Cytogenetic abnormalities, n							
t(15:17)	6	2	3	0	0	0	3
t(8:21)	3	4	0	1	0	0	0
inv(16)/t(16;16)	0	0	0	0	0	0	0
t(9;22)	0	0	0	0	0	0	3
Complex karyotype	0	2	0	0	0	0	0
Other karyotype	7	5	0	0	0	0	0
Normal karyotype	8	3	20	14	15	10	2
Not determined	0	0	3	1	0	0	8
Gene abnormalities, n							
PML/RARα	6	3	7	4	0	0	4
AML/ETO	3	6	4	2	0	0	0
BCR/ABL	0	2	0	0	0	0	3
IgH or TCR	1	0	0	0	0	0	0
Other gene	2	2	0	0	0	0	0
Not determined	12	3	15	10	15	10	9

AL, acute leukemia; HCR, hematological complete remission; MR, molecular remission; WBC, white blood cell; FAB, French-American-British; AML, acute myeloid leukemia; ALL, acute lymphoblastic leukemia; PML, promyelocytic leukema; RAR, retinoic acid receptor; ETO, eight-twenty-one; BCR, breakpoint cluster region; ABL, abelson; IgH, immunoglobulin heavy chain; TCR, T cell receptor.

**Table II:** Clinical characteristics of the control groups.

A, Healthy controls				

Characteristic	Training set		Test set	
Male:female, n	16:13		9:11	
Mean age, years (range)	39.6 (15–65)		39.2 (23–73)	

B, Benign hematological disorders				

Characteristic	Anemia	ITP	PNH	Aplastic anemia

Male:female, n	4:7	4:7	3:1	4:5
Mean age, years (range)	41.5 (12–78)	42.4 (14–75)	47.5 (35–63)	52.6 (43–68)

C, Solid tumor				

Characteristic	Liver	Breast	Lung	Colon

Male:female, n	5:1	0:9	5:3	3:4
Mean age, years (range)	48.7 (18–61)	51.3 (33–77)	51.8 (35–62)	53.0 (48–60)

ITP, idiopathic thrombocytopenia; PNH, paroxysmal nocturnal hemoglobinuria.

**Table III:** Reproducibility of mass spectra by magnetic beads and MALDI-TOF MS analysis.

	Within-run assays		Between-run assays	
		
m/z	MRI, %	CV, %	MRI, %	CV, %
2081.88	4.7	9.1	5.1	13.9
2861.24	0.8	11.0	0.8	17.7
3157.64	1.8	8.4	1.5	18.0
3240.01	0.7	7.5	0.6	15.7
4209.03	2.3	8.9	2.5	22.0
6633.21	2.4	9.0	2.3	22.3

MRI, mean relative intensity; CV, coefficient of variation; MALDI-TOF MS, matrix-assisted laser desorption/ionization-time of flight mass spectometry.

**Table IV:** Clinical characteristics of patients with newly-diagnosed AL who had complete records.

Clinical characteristics	Group A (n=18)	Group B (n=15)
Median age, years (range)	36 (17–73)	42 (17–75)
Gender ratio, M/F	11/7	7/8
FAB no. (%)		
M2	6 (33.3)	4 (26.7)
M3	5 (27.8)	5 (33.3)
M4	1 (0.06)	0
M5	0	2 (13.3)
ALL	6 (33.3)	4 (26.7)
Leukocytes >30g/l, n (%)	6 (33.3)	6 (40.0)
2-year survival, n (%)	9 (50.0)	4 (26.7)
Succumbed, n	9	11
Median survival, months	12	7

AL, acute leukemia; M, male; F, female; ALL, acute lymphoblastic leukemia.

## Abbreviations

AL: acute leukemia
CR: complete remission
AML: acute myeloid leukemia
ALL: acute lymphoblastic leukemia
MRD: minimal residual disease
MALDI-TOF MS: matrix-assisted laser desorption/ionization-time of flight mass spectrometry
PCR: polymerase chain reaction
MFC: multiparameter flow cytometry
APL: acute promyeloid leukemia
MR: molecular remission
ROC: receiver operating characteristic
AUC: area under the ROC curve
OS: overall survival

## References

[1] Falini B, Tiacci E, Martelli MP, Ascani S, Pileri SA, Swerdlow SH, Campo E, Harris NL, Jaffe ES, Pileri SA, Stein H (2008). Acute myeloid leukemia (AML) and related precursor neoplasms. WHO Classification of Tumors of Haematopoietic and Lymphoid Tissues.

[2] Pui CH, Relling MV, Downing JR (2004). Acute lymphoblastic leukemia. N Engl J Med.

[3] Kantarjian HM, O’Brien S, Smith TL, Cortes J, Giles FJ, Beran M (2000). Results of treatment with hyper-CVAD, a dose-intensive regimen, in adult acute lymphocytic leukemia. J Clin Oncol.

[4] Annino L, Vegna ML, Camera A, Specchia G, Visani G, Fioritoni G (2002). Treatment of adult acute lymphoblastic leukemia (ALL): long-term follow-up of the GIMEMA ALL 0288 randomized study. Blood.

[5] Cassileth PA, Harrington DP, Appelbaum FR, Lazarus HM, Rowe JM, Paietta E (1998). Chemotherapy compared with autologous or allogeneic bone marrow transplantation in the management to acute myeloid leukemia in first remission. N Engl J Med.

[6] Dworzak MN, Fröschl G, Printz D, Mann G, Pötschger U, Mühlegger N, Austrian Berlin-Frankfurt-Münster Study Group (2002). Prognostic significance and modalities of flow cytometric minimal residual disease detection in childhood acute lymphoblastic leukemia. Blood.

[7] Mortuza FY, Papaioannou M, Moreira IM, Coyle LA, Gameiro P, Gandini D (2002). Minimal residual disease tests provide an independent predictor of clinical outcome in adult acute lymphoblastic leukemia. J Clin Oncol.

[8] Campana D (2003). Determination of minimal residual disease in leukemia patients. Br J Haematol.

[9] San Miguel JF, Vidriales MB, López-Berges C, Díaz-Mediavilla J, Gutiérrez N, Cañizo C (2001). Early immunophenotypical evaluation of minimal residual disease in acute myeloid leukemia identifies different patient risk groups and may contribute to postinduction treatment stratification. Blood.

[10] Guerrasio A, Pilatrino C, De Micheli D, Cilloni D, Serra A, Gottardi E (2002). Assessment of minimal residual disease (MRD) in CBFbeta/MYH11-positive acute myeloid leukemias by qualitative and quantitative RT-PCR amplification of fusion transcripts. Leukemia.

[11] van Dongen JJ, Seriu T, Panzer-Grümayer ER, Biondi A, Pongers-Willemse MJ, Corral L (1998). Prognostic value of minimal residual disease in acute lymphoblastic leukaemia in childhood. Lancet.

[12] Schmidt HH, Strehl S, Thaler D, Strunk D, Sill H, Linkesch W (2004). RT-PCR and FISH analysis of acute myeloid leukemia with t(8;21)(p11;p13) and chimeric MOZ and CBP transcripts: breakpoint cluster region and clinical implications. Leukemia.

[13] Fiedler GM, Leichtle AB, Kase J, Baumann S, Ceglarek U, Felix K, Conrad T, Witzigmann H, Weimann A, Schütte C, Hauss J, Büchler M, Thiery J (2009). Serum peptidome profiling revealed platelet factor 4 as a potential discriminating Peptide associated with pancreatic cancer. Clin Cancer Res.

[14] Patz EF, Campa MJ, Gottlin EB, Kusmartseva I, Guan XR, Herndon JE (2007). Panel of serum biomarkers for the diagnosis of lung cancer. J Clin Oncol.

[15] Visintin I, Feng Z, Longton G, Ward DC, Alvero AB, Lai Y (2008). Diagnostic markers for early detection of ovarian cancer. Clin Cancer Res.

[16] Kornblau SM, Tibes R, Qiu YH, Chen W, Kantarjian HM, Andreeff M (2009). Functional proteomic profiling of AML predicts response and survival. Blood.

[17] Cui JW, Li WH, Wang J, Li AL, Li HY, Wang HX (2005). Proteomics-based identification of human acute leukemia antigens that induce humoral immune response. Mol Cell Proteomics.

[18] Liang T, Wang N, Li W, Li A, Wang J, Cui J (2010). Identification of complement C3f-desArg and its derivative for acute leukemia diagnosis and minimal residual disease assessment. Proteomics.

[19] Cao Y, Wang N, Ying X, Li A, Wang H, Zhang X, Li W (2009). BioSunMS: a plug-in-based software for the management of patients information and the analysis of peptide profiles from mass spectrometry. BMC Med Inform Decis Mak.

[20] Ng KL, Mishra SK (2007). De novo SVM classification of precursor microRNAs from genomic pseudo hairpins using global and intrinsic folding measures. Bioinformatics.

[21] Licastro F, Pedrini S, Ferri C, Casadei V, Govoni M, Pession A (2000). Gene polymorphism affecting alpha1-antichymotrypsin and interleukin-1 plasma levels increases Alzheimer’s disease risk. Ann Neurol.

[22] Bird PI (1999). Regulation of pro-apoptotic leucocyte granule serine proteinases by intracellular serpins. Immunol Cell Biol.

[23] Kloth JN, Gorter A, Fleuren GJ, Oosting J, Uljee S, ter Haar N (2008). Elevated expression of SerpinA1 and SerpinA3 in HLA-positive cervical carcinoma. J Pathol.

[24] Lai ML, Rizzo N, Liguori C, Zucca G, Faa G (1998). Alpha-1-antichymotrypsin immunoreactivity in papillary carcinoma of the thyroid gland. Histopathology.

[25] Estellés A, Gilabert J, Grancha S, Yamamoto K, Thinnes T, España F (1998). Abnormal expression of type 1 plasminogen activator inhibitor and tissue factor in severe preeclampsia. Thromb Haemost.

[26] Wang Y, Jiang H, Dai D, Su M, Martinka M, Brasher P (2010). Alpha 1 antichymotrypsin is aberrantly expressed during melanoma progression and predicts poor survival for patients with metastatic melanoma. Pigment Cell Melanoma Res.

